# Microbial Biopolymers: Trends in Synthesis, Modification, and Applications

**DOI:** 10.3390/polym15061364

**Published:** 2023-03-09

**Authors:** Shashi Kant Bhatia

**Affiliations:** 1 Department of Biological Engineering, College of Engineering, Konkuk University, Seoul 05029, Republic of Korea; shashibiotechhpu@gmail.com; 2 Institute for Ubiquitous Information Technology and Applications, Konkuk University, Seoul 05029, Republic of Korea

Microbes can act as a factory for the conversion of a variety of carbon and nitrogen sources into diverse kinds of intracellular and extracellular biopolymers, including polyhydroxyalkanoates (PHA) and exopolysaccharides (EPS), under different stress conditions [1,2]. Polyhydroxyalkanoates are intracellularly stored and serve as carbon and energy storage reserves. Almost 150 types of monomeric units have been reported and involved in the regulation of the physicochemical properties of the PHA [3]. A variety of microbes have recently been reported to produce PHA, such as *Bacillus mycoides*, *Halomonas cerina*, *Pseudomonas resinovorans*, etc. [4,5,6]. These biopolymers are biocompatible, biodegradable, and have different chemical and morphological properties that make them suitable for drug delivery, tissue engineering, packaging industry, and environmental applications [7]. Recent advances in molecular biology, transcriptomics, and metabolomics techniques have improved the understanding related to mechanisms and regulations involved in biopolymer synthesis [8,9]. A microbial system can be easily engineered and cultured under controlled conditions to produce different polymers. Biopolymers produced by microbial systems are rich in various functional groups that can be exploited further to modify the polymers for a variety of applications [10]. The production cost of biopolymers is the main challenge for their applicability on a commercial scale. Researchers are working on the utilization of diverse kinds of organic wastes, such as lignocellulosic waste, municipal waste, whey, paper, pulp industry waste, etc., as feedstocks for microbial fermentation. Biopolymer production from a microbial system is a clean and green approach that has recently become a popular topic worldwide, and it is considered a possible way to deal with plastic-based wastes, with extensive applications in the biotechnology sector. According to the Scopus database, approximately 1849 research and review articles have been published in the last few years (2018–2023). The top five countries involved in microbial biopolymer research are India (382), China (274), the United States (214), South Korea (122), and Brazil (135) (Figure 1).

To make biopolymer production economic, searching for microbes able to utilize cheap and abundantly available raw material is an important step. Galactose is a sugar found in marine algal biomass. Jung et al. screened 16 different *Halomonas* strains and reported a high PHB production (5.2 g/L) in *Halomonas cerina* with *Eucheuma spinosum* hydrolysate as a carbon source. *H. cerina* can survive in high-saline conditions and is able to accumulate PHA up to 72.41% *w*/*w* under unsterilized conditions [6]. In another study, researchers used cardboard hydrolysate as a carbon source for *Bacillus mycoides* and reported 56% *w*/*w* PHA accumulation [5]. Polyhydroxybutyrate (PHB) polymers have limited applications due to their brittle nature. To overcome this issue, researchers are working on the production of various copolymers of PHB. *Pseudomonas* sp. has the ability to produce medium-chain-length PHA (mcl-PHA) with improved properties. Jeon et al. used three different alkanes—n-octane, n-decane, and n-dodecane—as carbon sources, and the process resulted in mcl-PHA productionof 0.48 g/L, 0.27 g/L, or 0.07 g/L, respectively. The optimization of cultural conditions using statistical design, and the use of mixed alkane under optimized conditions at a 7 L fermentation scale produces 2.1 g/L mcl-PHA [4]. PHA recovery and downstream processing are also expensive processes. To make the PHA recovery process more economic, Novakova et al. developed a detergent-based PHA extraction method. This method includes the exposition of PHA-accumulating microbial biomass to hypotonic conditions at elevated temperatures in the presence of sodium dodecyl sulfate (SDS). This process resulted in a high PHA recovery with 99% purity [11]. The use of SDS is able to remove hydrophobic impurities and the further removal of SDS can be easily achieved using KCl as a precipitation method. To efficiently utilize feedstocks, Kang et al. developed a technology for PHA and hydrogen production, where oil was first extracted from spent coffee grounds and used as a feedstock for *Pseudomonas resinovorans* to produce PHA (1.6 g/L). Further, oil extracted residual spent coffee biomass was hydrolyzed to produce sugars and used as a carbon source for *Clostridium butyricum* DSM10702 to produce hydrogen (181.19 mL) [12]. Polyhydroxyalkanoates properties can be further improved by preparing copolymers or mixing and blending them with other natural and synthetic polymers. Jo et al. reported that copolymers with a 4HB content greater than 16% are non-crystalline in nature, while P(3HB-*co*-4HB) mixtures with the same 4HB content are crystalline. Due to this effect, the mixture has a higher melt viscosity and a lower tangent with better melt processing properties [13]. Polyhydroxyalkanoates have applications in the packaging industry, and PHA must be prepared as a foamed material with a porous structure. Zhang et al. used supercritical CO_2_ to prepare the foaming material of P(3HB-*co*-4HB) and reported that 4HB concentration plays an important role in foaming, while no foaming occurs beyond 50% 4HB content [14]. Phuegyod et al. prepared a poly(3-hydroxybutyrate-*co*-3-hydroxyvalerate) P(3HB-*co*-3HV) scaffold and compared its properties with other polymers for use in periodontal tissue engineering. The human gingival fibroblasts and periodontal ligament stem cells had a higher proliferation, healthy morphology, and better compatibility when cultured with P(3HB-*co*-3HV) as compared to other polymers [15]. To improve the mechanical strength of PHA, these polymers are blended with other materials such as plasticizers, which affect their biodegradability. Cho et al. prepared PHB blends with various plasticizers and studied the degradation of the blended materials. It was reported that a PHB blend with 10-20% tributyl citrate improves its mechanical properties as well as speeds up its degradation by *Microbulbifer* sp. SOL66 [16].

As the editor of this Special Issue, I have observed that the utilization of waste materials as feedstocks, the development of eco-friendly methods for PHA recovery, and the improvement of PHA properties by mixing are the current areas of interest among the published research and review articles. I am sure that this Special Issue will pique the interest of researchers in this area and provide readers with a broad and updated overview of this topic.

## Figures and Tables

**Figure 1:**
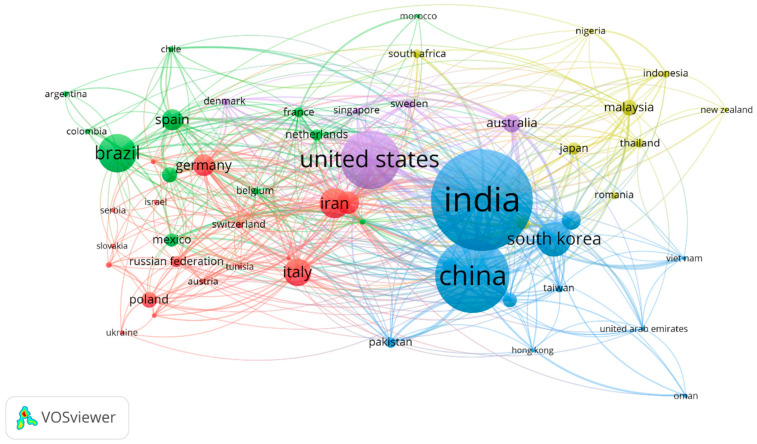
Co-occurrence mapping of publications related to microbial biopolymers (min. number of occurrence 5).

## References

[1] Bhatia S., Gurav R., Choi Y.-K., Choi T.-R., Kim H.-j., Song H.-S., Mi Lee S., Lee Park S., Soo Lee H., Kim Y.-G. (2021). Bioprospecting of exopolysaccharide from marine *Sphingobium yanoikuyae* BBL01: Production, characterization, and metal chelation activity. Bioresour. Technol..

[2] Jung H.-R., Choi T.-R., Han Y.H., Park Y.-L., Park J.Y., Song H.-S., Yang S.-Y., Bhatia S.K., Gurav R., Park H. (2020). Production of blue-colored polyhydroxybutyrate (PHB) by one-pot production and coextraction of indigo and PHB from recombinant *Escherichia coli*. Dyes. Pigments..

[3] Vu D.H., Wainaina S., Taherzadeh M.J., Åkesson D., Ferreira J.A. (2021). Production of polyhydroxyalkanoates (PHAs) by *Bacillus megaterium* using food waste acidogenic fermentation-derived volatile fatty acids. Bioengineered.

[4] Jeon J.-M., Park S.-J., Son Y.-S., Yang Y.-H., Yoon J.-J. (2022). Bioconversion of Mixed Alkanes to Polyhydroxyalkanoate by *Pseudomonas resinovornas*: Upcycling of Pyrolysis Oil from Waste-Plastic. Polymers.

[5] Abdelmalek F., Steinbüchel A., Rofeal M. (2022). The Hyperproduction of Polyhydroxybutyrate Using *Bacillus mycoides* ICRI89 through Enzymatic Hydrolysis of Affordable Cardboard. Polymers.

[6] Jung H.J., Kim S.H., Cho D.H., Kim B.C., Bhatia S.K., Lee J., Jeon J.-M., Yoon J.-J., Yang Y.-H. (2022). Finding of Novel Galactose Utilizing *Halomonas* sp. YK44 for Polyhydroxybutyrate (PHB) Production. Polymers.

[7] Bhatia S.K., Otari S.V., Jeon J.-M., Gurav R., Choi Y.-K., Bhatia R.K., Pugazhendhi A., Kumar V., Rajesh Banu J., Yoon J.-J. (2021). Biowaste-to-bioplastic (polyhydroxyalkanoates): Conversion technologies, strategies, challenges, and perspective. Bioresour. Technol..

[8] Lee H.S., Lee S.M., Park S.L., Choi T.-R., Song H.-S., Kim H.-J., Bhatia S.K., Gurav R., Kim Y.-G., Kim J.-H. (2021). Tung Oil-Based Production of High 3-Hydroxyhexanoate-Containing Terpolymer Poly(3-Hydroxybutyrate-co-3-Hydroxyvalerate-co-3-Hydroxyhexanoate) Using Engineered *Ralstonia eutropha*. Polymers.

[9] Jung H.-R., Yang S.-Y., Moon Y.-M., Choi T.-R., Song H.-S., Bhatia S.K., Gurav R., Kim E.-J., Kim B.-G., Yang Y.-H. (2019). Construction of Efficient Platform Escherichia coli Strains for Polyhydroxyalkanoate Production by Engineering Branched Pathway. Polymers.

[10] Emaimo A.J., Olkhov A.A., Iordanskii A.L., Vetcher A.A. (2022). Polyhydroxyalkanoates Composites and Blends: Improved Properties and New Applications. J. Compos. Sci..

[11] Novackova I., Kourilova X., Mrazova K., Sedlacek P., Kalina M., Krzyzanek V., Koller M., Obruca S. (2022). Combination of Hypotonic Lysis and Application of Detergent for Isolation of Polyhydroxyalkanoates from Extremophiles. Polymers.

[12] Kang B.-J., Jeon J.-M., Bhatia S.K., Kim D.-H., Yang Y.-H., Jung S., Yoon J.-J. (2023). Two-Stage Bio-Hydrogen and Polyhydroxyalkanoate Production: Upcycling of Spent Coffee Grounds. Polymers.

[13] Jo M., Jang Y., Lee E., Shin S., Kang H.-J. (2022). The Modification of Poly(3-hydroxybutyrate-co-4-hydroxybutyrate) by Melt Blending. Polymers.

[14] Zhang T., Jang Y., Lee E., Shin S., Kang H.-J. (2022). Supercritical CO_2_ Foaming of Poly(3-hydroxybutyrate-co-4-hydroxybutyrate). Polymers.

[15] Phuegyod S., Pramual S., Wattanavichean N., Assawajaruwan S., Amornsakchai T., Sukho P., Svasti J., Surarit R., Niamsiri N. (2023). Microbial Poly(hydroxybutyrate-co-hydroxyvalerate) Scaffold for Periodontal Tissue Engineering. Polymers.

[16] Cho J.Y., Kim S.H., Jung H.J., Cho D.H., Kim B.C., Bhatia S.K., Ahn J., Jeon J.-M., Yoon J.-J., Lee J. (2022). Finding a Benign Plasticizer to Enhance the Microbial Degradation of Polyhydroxybutyrate (PHB) Evaluated by PHB Degrader *Microbulbifer* sp. SOL66. Polymers.

